# Comparative Study of Flesh Quality, Blood Profile, Antioxidant Status, and Intestinal Microbiota of European Catfish (*Silurus glanis*) Cultivated in a Recirculating Aquaculture System (RAS) and Earthen Pond System

**DOI:** 10.3390/life13061282

**Published:** 2023-05-30

**Authors:** Cristian-Alin Barbacariu, Cristina Mihaela Rimbu, Marian Burducea, Lenuta Dirvariu, Liviu-Dan Miron, Razvan Stefan Boiangiu, Gabriela Dumitru, Elena Todirascu-Ciornea

**Affiliations:** 1 Research and Development Station for Aquaculture and Aquatic Ecology, “Alexandru Ioan Cuza” University, Carol I, 20A, 700505 Iasi, Romania; alin.barbacariu@uaic.ro (C.-A.B.); dirvariu.lenuta@gmail.com (L.D.); 2 Department of Public Health, Faculty of Veterinary Medicine, University of Life Sciences “Ion Ionescu de la Brad” Iaşi, Mihail Sadoveanu Alley 6-8, 700490 Iasi, Romania; lmiron@uaiasi.ro; 3 Faculty of Biology, “Alexandru Ioan Cuza” University, Carol I, 20A, 700505 Iasi, Romania; razvan.boiangiu@uaic.ro (R.S.B.); gabriela.dumitru@uaic.ro (G.D.); ciornea@uaic.ro (E.T.-C.)

**Keywords:** *Silurus glanis*, recirculating aquaculture system, earthen pond, growth performance, blood profile, proximate composition, oxidative status, microbiota

## Abstract

With the increasing demand for European catfish, traditional extensive growth methods in polyculture are no longer sufficient to meet market needs. Therefore, this study aimed to identify indicators for improving recirculating aquaculture system (RAS) technology by determining and comparing growth performance, flesh quality, blood profile, oxidative status, and intestinal microbiota parameters between fish cultivated in a RAS and an earthen pond. Results revealed that RAS-grown fish had a higher fat content compared to pond-grown fish, while no significant differences were found for growth parameters. Sensory analysis showed no significant difference in taste between the two groups. Blood composition analysis showed small differences. Oxidative status analyses showed higher catalase and glutathione peroxidase activities in RAS-grown fish and slightly higher superoxide dismutase activity in pond-grown fish. Microbial analysis showed differences in the intestinal microflora, with a higher total number of aerobic germs and anaerobic germs and a lower total number of sulfite-reducing clostridia in RAS-grown fish. This study provides valuable insights into the comparative performance of a RAS and a pond rearing system in European catfish production, potentially informing future growth technologies.

## 1. Introduction

European catfish (*Silurus glanis*) production is specific to countries such as Romania, Bulgaria, Croatia, the Czech Republic, France, Hungary, Poland, and Belgium. However, catfish production has declined in recent years in Romania (85.47 t in 2015, 48.025 t in 2016, 46.01 t in 2017, and 27.73 t in 2018), one of the countries where catfish are extensively raised in polyculture with common carp [1]. To optimize catfish production, numerous studies have been undertaken on various aspects of catfish aquaculture, such as artificial reproduction, larval rearing, and feeding [2,3,4,5,6,7,8,9]. To promote optimal growth in European catfish, it is recommended to maintain a controlled temperature of above 22 °C, provide fish feed with a protein value of 40–50% and a fat value of 10–12%, and maintain a feed conversion ratio of 0.8–1.5 kg of feed per kg of body weight [2,10,11]. However, European catfish are susceptible to various diseases caused by parasites such as *Ichthyophthirius multifiliis*, bacteria including Flexibacter and Flavobacterium, and viruses such as Iridovirus [12]. To prevent *Ichthyophthirius multifiliis* infections, prophylactic administration of dimetridazole is recommended in the early days of feeding [2]. Furthermore, the biochemical composition of European catfish meat is influenced by rearing technology, water temperature, and feed, resulting in meat with approximately 77.9% water, 17.3% protein, 3.7% lipids, and 0.1% carbohydrates [13,14,15,16,17]. Nonetheless, there is currently limited knowledge regarding the effects of various cultivation technologies, particularly a recirculating aquaculture system (RAS), on catfish growth performance, blood profile, flesh proximate composition, oxidative status, and intestinal microbiota. As the demand for catfish consumption continues to rise, it is imperative to gain a deeper understanding of how different cultivation methods impact catfish physiology and meat quality. A RAS has become increasingly popular in recent years, particularly for the production of high-value fish species. According to a 2020 EUMOFA document [18], the top five species cultivated in a RAS in 2018 accounted for 95% of total production, with rainbow trout being the most farmed species at 56%. North African catfish, European eel, Atlantic salmon, and Senegalese sole were the next most commonly farmed species. Notably, European catfish were not among the top five species, indicating a need for further research and development of RAS technology to make it economically feasible for this species. A RAS offers several advantages over traditional pond systems, including improved water quality control, reduced water usage, and higher fish density [19]. However, one challenge associated with a RAS is the potential for high levels of oxidative stress due to the increased fish density and limited water exchange. Oxidative stress can have negative effects on fish growth, immune function, and overall health [20]. Therefore, it is important to investigate the impact of a RAS on the oxidative status of European catfish and identify strategies to mitigate any negative effects. Another important aspect to consider in European catfish aquaculture is the role of intestinal microbiota. The gut microbiota plays a vital role in nutrient digestion and absorption, immune system development, and the overall health of the fish. Recent studies have demonstrated the potential for manipulating the gut microbiota to improve fish growth, disease resistance, and overall performance [21]. Therefore, understanding the effects of a RAS and pond production systems on the gut microbiota of European catfish could provide valuable insights into optimizing production and improving fish health. This study aims to compare the effects of a RAS and a pond rearing system on the growth, flesh quality, blood profile, oxidative status, and intestinal microbiota of European catfish, *Silurus glanis*.

## 2. Materials and Methods

### 2.1. Experimental Protocol

The experiment was conducted over a three-year period (2019–2022) at the Research and Development Station for Aquaculture and Aquatic Ecology, “Alexandru Ioan Cuza” University in Iasi, Romania.

In 2019, a family of European catfish broodstock was selected for natural propagation in a metal tank with a water volume of 75 m^3^. The resulting offspring were introduced into the earthen pond and reared in a semi-intensive production system in polyculture with common carp (*Cyprinus carpio*) that served as sanitary fish. After one year, the offspring were split into two groups: one was introduced into the RAS (*n* = 10; 4 replicates), and the other was reintroduced into the earthen pond and monitored for a period of two years (*n* = 40).

### 2.2. Productivity Indices

The fish were weighed at the beginning and at the end of the experiment. The following parameters were calculated [22]:

IBW—initial body weight (g);

FBW—final body weight (g);

WG—weight gain (%) = ((FBW − IBW)/IBW) × 100;

FCR—feed conversion ratio (g/g) = feed intake (g)/WG;

RGR—relative growth rate (g/g day^−1^) = WG/days of experiment/IBW;

SGR—specific growth rate (% day^−1^) = ((ln FBW − ln IBW)/days of experiment) × 100;

PER—protein efficiency ratio = WG/total protein;

Fulton’s condition factor (CF) = [FBW (g)/(SL (cm)^3^)] × 100, where SL = standard body length;

Hepatosomatic index (HSI) = [liver weight (g)/body weight (g)] × 100; 

Viscerosomatic index (VSI) = [visceral weight (g)/body weight (g)] × 100;

Survival Rate (%) = (number of surviving fish/number of initial fish) × 100

### 2.3. Physico-Chemical Parameters of Water

During the entire experimental period, the physico-chemical parameters of the fish water were monitored daily to ensure that they remained within the optimal range for catfish growth. The water quality in both the recirculating system and earthen pond was carefully maintained to prevent stress on the fish. The European catfish is known to be a hardy species, tolerant of varying environmental conditions. Optimal temperatures for growing European catfish range between 20 and 24 °C [23]. To monitor water quality, dissolved oxygen content, temperature, and pH were measured daily using a HQ30d flexiparameter and a Hach model HQ11d digital pH meter, respectively. The levels of nitrates, nitrites, ammonia, ammonium, and phosphorus were determined using a Hanna Vis Iris HI-801 spectrophotometer. The results of these measurements are shown in Table 1.

### 2.4. The Proximate Flesh Composition

The proximate flesh composition of fresh and cooked flesh was analyzed with the DA 7250 NIR, Perten Instruments, Hagersten, Sweden. This device uses near-infrared light to quickly and accurately measure the moisture, fat, protein, ash, collagen, salt, and collagen-free protein of the fish flesh without special preparation. Briefly, the meat samples were homogenized with a meat grinder, placed in a tray, and analyzed. The analysis report is obtained automatically in 6 s. The results are expressed in percent as they are based on pre-existing calibrations included in the device software.

### 2.5. Blood Parameters

To obtain blood samples, the fish were anesthetized using 0.03 mL/l clove oil [24]. The blood was then collected through a heart puncture. The blood samples were analyzed using the MNCHIP Pointcare V2 Analyzer to determine the biochemical and hematological profiles. The following biochemical indices were measured: ALB—albumin; TP—total proteins; GLO—globulins; A/G—ratio between albumin and globulins; Ca—calcium; GLU—glucose; BUN—urea; P—phosphorus; AMY—amylase; CHOL—cholesterol; ALT—ala-nine aminotransferase; TBIL—total bilirubin; ALP—alkaline phosphatase; CRE—creatinine; BUN/CRE—ratio between urea and creatinine; and CK—creatine kinase.

Hematological indices were also determined, including: WBC—white blood cell count; LYM—lymphocyte count; MON—monocyte count; NEU—neutrophil count; EOS—eosinophil count; BAS—basophil count; LYM—lymphocyte percentage; MON—monocyte percentage; NEU—neutrophil percentage; EOS—eosinophil percentage; BAS—basophil percentage; RBC—red blood cell count; HGB—hemoglobin concentration; HCT—hematocrit percentage; MCV—mean corpuscular volume; MCH—mean corpuscular hemoglobin; MCHC—mean corpuscular hemoglobin concentration; RDWc—red cell distribution width coefficient of variation; RDWs—red cell distribution width standard deviation; PLT—platelet count; MPV—mean platelet volume; PCT—plateletcrit percentage; PDWc—platelet distribution width coefficient of variation; and PDWs— platelet distribution width standard variation.

### 2.6. Oxidative Status Assessment

The fish were euthanized with a 2% clove oil solution, and muscle, liver, and intestine tissue samples were precisely dissected and collected for oxidative status assessment. The tissue samples were homogenized in an ice-cold potassium phosphate buffer solution of 0.1 M, KCl 1.15%, pH 7.4, in a ratio of 1:10 (*w*/*v*). The homogenates were centrifuged for 20 min at 3000 rpm at 4 °C, and the supernatants were further used to measure the activities of superoxide dismutase (SOD), catalase (CAT), and glutathione peroxidase (GPX). Additionally, they were used to determine the content of reduced glutathione (GSH), malondialdehyde (MDA), and carbonylated proteins according to the methods described in Boiangiu et al. (2023) [25]. The SOD, CAT, and GPX activities and the levels of GSH, MDA, and carbonylated proteins were normalized to the total content of soluble proteins measured by the Bradford method (1976) [26].

### 2.7. Sensory Analysis by Triangle Test

The main objective of the triangle test is to determine if there are differences in terms of taste between the European catfish grown in the RAS and the one from the earthen pond. For this, two batches of catfish meat were cooked, one from the RAS and the other from the pond. Thirteen assessors received three coded samples, two identical and one different, and were asked to identify the different sample. The samples were prepared according to the scheme: 2 samples of ABB, 2 samples of AAB, 2 samples of ABA, 2 samples of BAA, 2 samples of BBA, and 2 samples of BAB. A represents catfish meat from the pond, and B is from the RAS. The samples were randomly distributed to the assessors. A statistical analysis of the results was performed by comparing the number of correct identifications with the number expected to be obtained by chance alone. To be statistically significant, the number of correct identifications corresponding to 13 assessors must be 8 for the 5% level and 9 for the 1% level [27].

### 2.8. Intestinal Microbiota

To analyze the effect of the cultivation system on the intestinal microbiota in *Silurus glanis*, gut content samples were collected from 9 fish from each batch. The entire gastrointestinal mass was collected by dissection, and the intestinal contents were harvested, distributed, and weighed under sterile conditions to obtain samples weighing 1 g. Four microbiological indicators, representative of the intestinal microbiota, were studied: the total number of aerobic germs (TNA/g intestinal content), the total number of anaerobic germs (TNAN/g intestinal content), the total number of sulfite-reducing clostridia (TNSRC/g intestinal content), and the total number of enterobacteria (TNE/g intestinal content). The working stages and culture media used were specific for each microbiological indicator [28,29]. The results were expressed in colony-forming units per gram of intestinal content (cfu/g).

### 2.9. Statistical Analysis

To assess the normality of the growth parameters, flesh proximate composition, blood profile, and logarithmically transformed microbiota data, the Shapiro-Wilk test was conducted. The normally distributed data were then analyzed using the Independent *t*-test, while the non-normal data were analyzed using the Mann-Whitney U test using SPSS software version 21 (IBM Corp., Armonk, NY, USA). The results were reported as means ± standard errors (S.E.M.).

The oxidative status results were analyzed by two-way ANOVA followed by Šídák’s multiple comparisons tests using GraphPad Prism software v9.3.1 (La Jolla, CA, USA). The significant differences were considered when *p* < 0.05 and the values were expressed as means ± S.E.M.

## 3. Results

### 3.1. Growth Performance Parameters

Table 2 displays the growth performance parameters of European catfish, which include initial weight, final weight, weight gain, condition factor, visceral somatic index (VSI), and hepatosomatic index (HSI). Although there were minor variations in these parameters between the fish grown in the recirculating aquaculture system (RAS) and those grown in the earthen pond, no statistically significant differences were observed, except for VSI and HSI. However, it is worth noting that RAS-grown fish showed a better weight gain (1232%) compared to pond-grown fish (1150%), which could result in higher yields in the RAS system.

Feed use efficiency parameters of European catfish cultivated in the RAS during the two years of the experiment are shown in Table 3. It can be observed that SGR and RGR decreased in the second year of the experiment, while FCR and PER increased.

### 3.2. Proximate Composition of Flesh

Table 4 presents the proximate composition of fresh and cooked European catfish flesh. In terms of fresh flesh composition, fish grown in the RAS had a significantly higher fat content and a significantly lower moisture and ash content compared to those grown in the earthen pond. However, there were no significant differences observed in the protein, collagen, or salt parameters. It is worth noting that there were no significant differences observed in any parameter for cooked meat.

### 3.3. Sensory Analysis

Table 5 shows the results of the sensory analysis of European catfish meat. Only 6 of the 13 assessors correctly identified the different sample. Since, for the result to be significant at the 5% level, 8 correct answers were necessary, the sensory analysis shows that there are no differences in the taste of the meat regardless of the cultivation method.

### 3.4. Blood Biochemical and Hematological Parameters

Table 6 presents the biochemical composition of the blood of European catfish. Small and insignificant variations were observed for ALB, TP, GLO, A/G, Ca, GLU, BUN, P, AMY, CRE, and BUN/CRE parameters between fish grown in the RAS and the pond. Although the cholesterol content was higher in RAS-grown fish, the difference was not statistically significant. However, ALT was significantly higher in RAS-grown fish. In contrast, TBIL and CK parameters were significantly higher in pond-reared fish.

Table 7 presents the hematological parameters of the blood in European catfish. WBC and NEU were significantly higher in RAS-grown fish, whereas HGB and MCH were significantly higher in pond-reared fish. There were no significant differences observed in the rest of the parameters.

### 3.5. Oxidative Status

**Figure 1:**
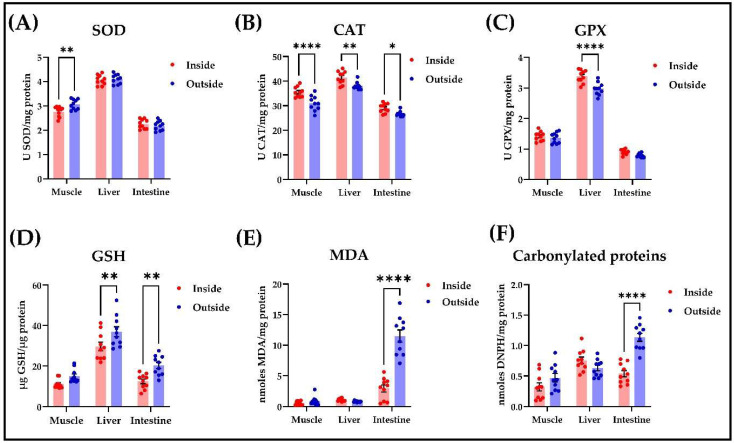
The influence of the growth environment (inside—RAS, outside—pond) on oxidative status determined in muscle, liver, and intestine tissues of *Silurus glanis*. The enzymatic parameters consisted of measuring (**A**) SOD-, (**B**) CAT-, and (**C**) GPX-specific activities, while the non-enzymatic parameters consisted of estimating the levels of (**D**) GSH, (**E**) MDA, and (**F**) carbonylated proteins. The values are expressed as means ± S.E.M. (*n* = 10). A two-way ANOVA analysis revealed overall significant differences between the experimental groups in (**A**) F(2,54) = 447.7, *p* < 0.01; (**B**) F(2,54) = 156.2, *p* < 0.0001; (**C**) F(2,54) = 1199, *p* < 0.0001; (**D**) F(2,54) = 89.47, *p* < 0.01; (**E**) F(2,54) = 117.1, *p* < 0.0001; and (**F**) F(2,54) = 28.41, *p* < 0.0001. For Šídák’s multiple comparisons analysis: **** *p* < 0.0001; ** *p* < 0.01; * *p* < 0.05. *n* = 3.

The analysis of biochemical indices in muscle, liver, and intestinal tissue samples revealed that the growth conditions definitely influence the oxidative status (Figure 1). Thus, with regard to the SOD activity (Figure 1A), slightly increased values are noted in the case of the sample grown in the conditions of the external environment (pond) in all types of tissues sampled, with maximum values in the liver tissue, the central seat of the metabolism of substances and energy, and a significant increase in muscle according to Šídák’s multiple comparisons (*p* < 0.01). In CAT and GPX, a different influence of growth conditions is observed in the sense that higher activity values were recorded for all types of tissue in the sample grown indoors in the RAS compared to the batch originating from the pond (Figure 1B,C). According to Šídák’s multiple comparisons, the highest increase in CAT activity was obtained in muscle (*p* < 0.0001), followed by liver (*p* < 0.01) and intestine (*p* < 0.05). Once more, in the case of GPX, we can notice the net difference in activity between liver tissue and intestinal tissue, respectively, with the only significant increase in the case of liver tissue (*p* < 0.0001). GSH is distinguished by slightly higher concentrations in the samples from the pond; the difference between the batch grown in natural conditions and the one in the RAS is, however, slightly significant according to Šídák’s multiple comparisons test (*p* < 0.01) in the liver and intestine (Figure 1D). With regard to the concentration of MDA and carbonylated proteins (Figure 1E,F), we can emphasize, on the one hand, the net difference between the muscle and liver tissue samples in comparison with the intestinal tissue samples, and on the other hand, the strongly significant difference, according to Šídák’s multiple comparisons test (*p* < 0.0001), which manifests itself in the case of intestinal tissue as a result of the influence exerted by environmental factors.

### 3.6. Intestinal Microbiota

**Figure 2:**
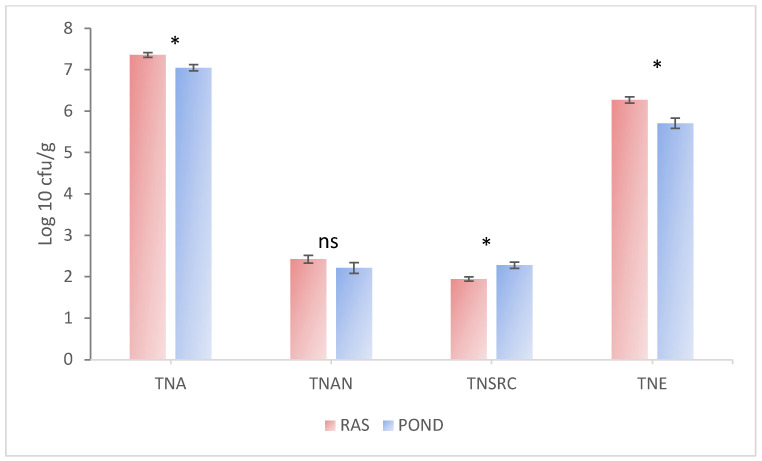
Logarithmic abundance of intestinal microbiota in European catfish from the recirculating aquaculture system (RAS) and earthen pond. Total number of aerobic germs (TNA), total number of anaerobic germs (TNAN), total number of sulfite-reducing clostridia (TNSRC), and total number of enterobacteria (TNE). * Significant differences for *p* ˂ 0.05; ns—no significant differences (*p* > 0.05). According to the Independent *t* test, *p*-values for TNA, TNAN, and TNSRC were 0.004, 0.207, and 0.002, respectively. The *p*-value for TNE was 0.001 according to the Mann-Whitney U test. *n* = 9.

Figure 2 presents the four microbiological indicators representative of the intestinal microbiota of *Silurus glanis*: total number of aerobic germs (TNA), total number of anaerobic germs (TNAN), total number of sulfite-reducing clostridia (TNSRC), and total number of enterobacteria (TNE). The analysis of the logarithmic transformed values showed significant differences between the two rearing systems for TNA, TNSRC, and TNE, while no significant differences were recorded for TNAN. Higher TNA and NTE and lower TNSRC in RAS-grown fish were found.

## 4. Discussion

The development of a recirculating aquaculture system (RAS) has provided many benefits to the aquaculture industry, such as the ability to control cultivation conditions and predict production yields, more efficient use of water and space resources, and reduced environmental impact. However, RAS technology is inherently complex and requires large initial investment costs and specialized personnel to operate the system, which can be a drawback for some operators [19]. The aim of this study was to compare the effects of two cultivation systems, RAS versus pond, on the growth, flesh quality, biochemical and hematological profiles of the blood, oxidative status, and intestinal microbiota of *Silurus glanis* in the climatic conditions of north-eastern Romania. Regarding production parameters, although no statistically significant differences were observed, weight gain was greater in the RAS group (1232%) compared to the pond group (1150%), which could indicate higher final production and economic value in the RAS. In fact, *Silurus glanis*, a freshwater species commonly known as the European catfish or Wels catfish, can achieve high production rates in intensive aquaculture systems. For instance, Mongirdas and Kusta (2006) [30] reported production rates of up to 30 kg/m^3^ in a RAS. Furthermore, other parameters such as VSI, processing fillet yield, and fat were higher in a RAS when compared to a flow-through system, according to Zdenek et al. (2015) [31]. The growth performance of *Silurus glanis* can be influenced by various factors, including the rearing system, water temperature, stocking density, and feeding regime. In recent years, the cultivation of *Silurus glanis* in a RAS has gained popularity due to its potential for high production rates and reduced environmental impact. A RAS facilitates the efficient use of water and the control of water quality parameters, resulting in improved growth rates and reduced mortality. However, the cultivation of European catfish in a RAS also has challenges, such as cannibalism and susceptibility to infestation with *Ichthyophthirius multifiliis* during the fry-to-fingerling stage, requiring a prophylactic dimetridazole treatment of 56 mg/kg for 10 days after feeding initiation [2]. *Silurus glanis* is a highly sought-after species in the European market, prized for its delicate flavor and texture. Its demand remains consistently strong, with the species commanding higher prices than other freshwater fish in several markets. However, in an earthen pond, the production rate of this fish is relatively low. For instance, at a density of 30 fish/ha, the first-year culture yields up to 18.4 kg/ha, while in the second year, a density of 130 fish/ha produces 61.3 kg/ha, and in the third year, a density of 70 fish/ha produces 36.3 kg/ha [32]. The quality of the meat is directly influenced by the cultivation conditions, and in this study, the proximate composition analyses showed that RAS-grown fish had a significantly higher fat content. This could be attributed to the feed composition being high in proteins and fat (45% and 12%, respectively) and a reduced swimming space for the fish. Jankowska et al. (2007) [33] also reported a high fat content in European catfish cultured in a RAS. However, the proximate composition of the cooked meat did not show significant differences between the two rearing systems in this study. Although the fat content was higher in the RAS group, sensory analysis of meat taste using the triangle test did not show significant differences between the two rearing systems, favoring cultivation in the RAS.

The blood’s biochemical composition and hematological profile provide valuable information about the fish’s health status and the presence of disturbing factors in the living environment [34]. This study found slight variations in both blood biochemical and hematological profiles, indicating that the growth system, particularly the RAS, causes a reduced level of stress. For example, the concentration of alanine aminotransferase, which is involved in amino acid metabolism, was significantly higher in RAS-reared fish, with values twice as high as those observed in pond-reared fish. This finding suggests a hepatic cell injury or increased synthesis of the enzyme by the liver, given that it is predominantly found in the liver, plasma, and various tissues. Conversely, total bilirubin (TBIL), alkaline phosphatase (ALP), and creatine kinase (CK) levels were significantly higher in pond-reared fish, indicating a stress response to the growing conditions. According to Hastuti et al. (2019) [35], elevated levels of bilirubin, which is the final product of hemolysis, can indicate an increase in haem breakdown and a decrease in total bilirubin intake by the liver, leading to yellow pigmentation and a condition known as jaundice. In the case of *Clarias gariepinus*, TBIL values were recorded as 7.9 in those affected by jaundice and 0.8 mg dL^−1^ in healthy fish. However, considering the fact that the values of European catfish grown in the earthen pond in this study were 1.86, which is close to the values of healthy fish from the study by Hastuti et al. (2019) [35], we can conclude that although there are significant differences compared to the RAS, there is no cause for concern. In contrast, the CK values, which are involved in adenosine triphosphate homeostasis and considered a biomarker for the presence of environmental stressors, such as drugs or pesticides that affect the muscles [36], were approximately three times higher in the pond-reared fish, suggesting the possible presence of some toxic compounds in the water. Regarding blood hematological parameters, significant variations were observed in the case of blood cells with immunological functions, such as white blood cells and neutrophils, which were four times higher in RAS-reared fish, while hemoglobin and mean hemoglobin volume were slightly higher in pond-reared fish. It is important to note that several factors can influence the blood biochemistry and hematology of *Silurus glanis*, including age, sex, reproductive status, diet, water quality, and disease status [37]. In this study, water quality and feed in the two rearing systems were the factors that influenced the parameters mentioned above.

Water recirculation systems are a reliable technological source being used in aquaculture to intensify fish production. Depending on the species, it is known that one of the limiting factors in such systems is the oxygen concentration [30,38,39]. At the same time, the fish’s defensive antioxidant system can be influenced by the oxygen concentration and toxins in the water and the fish’s temperature, age, feeding behavior, stress, etc. Many studies carried out in fish have focused on toxicological aspects and less on the relationship that exists between the oxidative cycle and seasonal variations or between the oxidative cycle and environmental conditions (recirculating system and earthen pond) [40]. Fish are frequently exposed to episodes of environmental and physiological hypoxia and are likely to produce high levels of reactive oxygen species (ROS), such as superoxide radical (O_2-_), hydrogen peroxide (H_2_O_2_), and hydroxyl radical (OH-), as a result of oxidative metabolism during physiological stress or the post-stress recovery period [41,42]. The excessive accumulation of these derivatives of harmful oxygen causes oxidative stress, whose direct effect is the peroxidation of important macromolecules. Thus, to reduce the negative effects of ROS, fish and other vertebrates possess an antioxidant defense system that includes enzymatic and non-enzymatic mechanisms. In addition, antioxidant enzymes respond differently to the action of stressors; thus, in this sense, to indicate the total oxyradical scavenging capacity, an overview of all these biochemical markers is necessary [43,44]. Regarding our results, the detected values differ, on the one hand, depending on the growth conditions and, on the other hand, depending on the type of analyzed tissue. Higher values were recorded, in general, in the liver tissue, which is likely due to the close connection with the intense metabolism that occurs at the level of this organ. In this aspect, our data is correlated with data from the literature in this field [45,46]. In addition, other studies [47,48,49] show the involvement of the lipid level and some vitamins in the daily diet in the defense of the antioxidant system and oxidative status in fish, with the soluble starch being involved in reducing the susceptibility of the fish to oxidation and potentially enhancing the growth rate.

From a microbiological point of view, higher logarithmic values were obtained in *Silurus glanis* RAS-grown than in pond-grown, except for the total number of sulfate-reducing clostridia, where the logarithmic value was lower (log10 1.9) than that in pond-grown (log10 2.3). Clostridia are commonly found in soil, aquatic environments, and the intestines of animals [50]. These microorganisms form endospores that allow bacteria to survive in almost any habitat, terrestrial or aquatic, by waiting for favorable growth conditions [51]. There is little bibliographic information on the density of sulfite-reducing Clostridia in the gut microbiome of freshwater fish, although these anaerobic microorganisms (including *Clostridium perfringens*), together with aerobic microorganisms from the Enterobacteriaceae family (including *Escherichia coli*), are considered fecal indicators for assessing the microbiological quality of water in the EU and UK [52,53,54]. Some authors hypothesized that the presence and high density of *Clostridium perfringens* (a sulfite-reducing species) in the gastrointestinal mass of fish could be a consequence of adaptation to the specific gut conditions of poikilotherms, giving fish the quality of a primary source of water contamination [55]. Since the effect of diet on the gut microbiota is known in humans and other species, we can infer that the population of sulfite-reducing clostridia in the gut of *Silurus glanis* fish from the RAS group was affected by the diet. Different dietary fats have different effects on the composition of the gut microbiota due to their different fatty acid profiles [56]. The results of our study are in agreement with other studies that have shown that members of the phylum Firmicutes (mainly unclassified Clostridiales and *Clostridium sensu stricto*) and Proteobacteria (Gammaproteobacteria) are the dominant taxa in the intestinal contents of different fish species [29,57]. It is well known that the gut microbiota plays an important role in host health by interacting with various physiological processes [58]. As long as the diet is balanced and provides nutritional benefits, the overall health of the fish does not suffer, and there are no economic losses. The cultivation technology of *Silurus glanis* can be improved by adjusting the environmental and feeding conditions to meet the species’ needs. Such an improvement could encourage farmers to increase production, especially given the high demand for this species. *Silurus glanis* is valued not only for its taste but also for its nutritional qualities, including a favorable fatty acid profile and better sanogenic indices [59]. Compared to other commonly consumed freshwater fish species, catfish has a significant proportion of polyunsaturated fatty acids (PUFA) and superior sanogenic indices, which suggests that it is of better quality [59]. Based on the results of our study, we recommend the adoption of RAS technology in European catfish aquaculture. The use of a RAS can lead to higher production levels without compromising the nutritional quality or welfare of the fish. Additionally, a RAS can offer a sustainable alternative to a traditional pond rearing system, as it consumes less water and can minimize environmental impacts.

## 5. Conclusions

Overall, the results of this study suggest that RAS technology has the potential to increase the yield of *Silurus glanis* in aquaculture as a sustainable alternative to cultivation in an earthen pond. Additionally, the small and insignificant differences in cooked meat composition and sensory analyses suggest that the fish raised in either system are comparable in terms of nutritional value and taste. Furthermore, the study found no adverse impact on the intestinal microbiota of the fish in either system. However, it is important to note that RAS cultivation is not without challenges, including maintaining water quality parameters within optimal ranges. Overall, this study provides valuable insights into the potential benefits and challenges of RAS technology for the cultivation of *Silurus glanis*.

## Figures and Tables

**Table 1:** Physico-chemical parameters of water.

		RAS			Earthen Pond		
Year	Parameters	Min.	Max.	Mean	Min.	Max.	Mean
First year	Temperature (°C)	16.9	25.50	20.70	16.40	28.10	22.50
	pH	8.10	8.30	8.20	8.20	8.50	8.35
	O_2_ (mg/L)	7.11	8.90	8.00	4.63	8.08	6.35
	NO_3_^−^ (mg/L)	7.20	26.40	16.8	0.50	1.30	0.90
	NO_2_^−^ (mg/L)	0.00	0.040	0.02	0.10	0.30	0.20
	NH_3_^+^ (mg/L)	0.00	0.06	0.03	0.01	0.17	0.09
	NH_4_^+^ (mg/L)	0.00	0.80	0.40	0.02	0.19	0.10
	P total (mg/L)	0.70	1.26	0.98	0.20	0.43	0.31
Second year	Temperature (°C)	15.2	25.8	20.5	15.60	27.80	21.70
	pH	8.10	8.20	8.15	8.30	8.40	8.35
	O_2_ (mg/L)	6.92	9.42	8.17	4.19	8.33	6.26
	NO_3_^−^ (mg/L)	2.60	11.60	7.10	0.00	6.60	3.30
	NO_2_^−^ (mg/L)	0.01	0.07	0.04	0.05	0.15	0.10
	NH_3_^+^ (mg/L)	0.04	0.15	0.09	0.01	0.09	0.05
	NH_4_^+^ (mg/L)	0.05	0.17	0.11	0.00	0.11	0.05
	P total (mg/L)	0.10	0.06	0.08	0.10	0.40	0.25

**Table 2:** Growth parameters of European catfish from the recirculating aquaculture system and earthen pond.

Aquaculture System	IBW (g)	FBW (g)	WG (g)	CF	VSI	HSI	Survival Rate (%)
RAS	174.38 ± 16.34	2233.5 ± 101.61	1232.09 ± 73.31	0.72 ± 0.03	6.49 ± 0.57	2.69 ± 0.19	100
Earthen Pond	212.05 ± 10.42	2598.1 ± 141.89	1150.95 ± 86.99	0.76 ± 0.03	4.55 ± 0.35	2.04 ± 0.12	100
Independent *t* test	0.68	0.51	0.485	-	0.009	0.011	1
Mann-Whitney U	-	-	-	0.143	-	-	-

IBW—initial body weight; FBW—final body weight; WG—weight gain; CF—condition factor; VSI—viscero somatic index; and HSI—hepato somatic index. *n* = 20.

**Table 3:** Feed efficiency parameters of European catfish cultivated in the RAS.

Year	SGR (% Day^−1^)	RGR (g/g Day^−1^)	FCR (g/g)	PER (g/g)
First year	9.57 ± 1.03	87.94 ± 12.29	1.58 ± 0.16	1.66 ± 0.28
Second year	5.13 ± 1.53	73.37 ± 16.38	1.66 ± 0.29	1.85 ± 0.39

SGR—specific growth rate; RGR—relative growth rate; FCR—feed conversion ratio; and PER—protein efficiency ratio.

**Table 4:** Proximate composition of European catfish flesh from the recirculating aquaculture system and earthen pond.

Fresh							
Rearing System	Fat (%)	Moisture (%)	Protein (%)	Collagen (%)	Salt (%)	Ash (%)	Collagen Free Protein (%)
RAS	14.08 ± 1.64	68.63 ± 1.15	16.86 ± 0.21	0.38 ± 0.08	0.26 ± 0.10	1.99 ± 0.14	-
Earthen Pond	7.11 ± 0.90	71.86 ± 0.64	16.63 ± 0.20	0.34 ± 0.07	0.54 ± 0.14	2.58 ± 0.10	-
Independent *t* test	0.001	0.020	0.436	-	-	0.001	-
Mann—Whitney U	-	-	-	0.979	0.232	-	-
Cooked							
Rearing system	Fat (%)	Moisture (%)	Protein (%)	Collagen (%)	Salt (%)	Ash (%)	Collagen free protein (%)
RAS	10.61 ± 0.05	66.69 ± 0.10	19.94 ± 0.16	1.4 ± 0.02	0.75 ± 0.06	1.50 ± 0.06	19.55 ± 0.43
Earthen Pond	10.48 ± 1.87	66.02 ± 1.21	20.92 ± 0.53	1.24 ± 0.09	0.91 ± 0.15	2.00 ± 0.23	22.42 ± 0.98
Independent *t* test	-	-	0.153	0.160	0.379	0.104	0.055
Mann-Whitney U	0.7	0.7	-	-	-	-	-

*n* = 6.

**Table 5:** Sensory evaluation of the taste of European catfish meat by the triangle test.

Evaluator	Sample	Result
1	AAB	0
2	AAB	0
3	ABA	0
4	BAA	0
5	BBA	1
6	BBA	0
7	BAB	0
8	ABB	1
9	ABB	0
10	ABA	1
11	BAB	1
12	ABB	1
13	ABB	1

A represents catfish meat from the earthen pond, and B is from the RAS. 1 represents correct identification, and 0 is wrong identification.

**Table 6:** Biochemical parameters of European catfish blood from the recirculating aquaculture system and earthen pond.

Parameter	RAS	Earthen Pond	Independent *t* test	Mann-Whitney U
ALB (g/dL)	1.57 ± 0.03	1.53 ± 0.09	0.742	-
TP (g/dL)	3.67 ± 0.24	3.40 ± 0.15	0.402	-
GLO (g/dL)	2.10 ± 0.21	1.87 ± 0.09	0.360	-
A/G	0.77 ± 0.07	0.83 ± 0.03	-	0.4
Ca (mg/dL)	9.60 ± 0.55	10.30 ± 0.26	0.316	-
GLU (mg/dL)	62.33 ± 7.22	65.33 ± 9.94	0.819	-
BUN (mg/dL)	1.09 ± 0.00	1.09 ± 0.00	1.00	-
P (mg/dL)	7.47 ± 0.73	9.53 ± 0.52	0.084	-
AMY (U/L)	13.00 ± 4.04	13.33 ± 3.38	0.953	-
CHOL (mg/dL)	392.00 ± 51.00	274.67 ± 31.32	-	0.1
ALT (U/L)	124.00 ± 23.86	52.00 ± 7.00	0.044	-
TBIL (mg/dL)	0.43 ± 0.15	1.86 ± 0.25	0.008	-
ALP (U/L)	279.33 ± 3.33	347.00 ± 16.56	-	0.1
CRE (mg/dL)	0.69 ± 0.09	0.83 ± 0.07	0.288	-
BUN/CRE	1.65 ± 0.25	1.33 ± 0.10	0.294	-
CK (U/L)	584.33 ± 150.67	1850.00 ± 0.00	0.001	-

ALB—albumin; TP—total proteins; GLO—globulins; A/G—ratio between albumin and globulins; Ca—calcium; GLU—glucose; BUN—urea; P—phosphorus; AMY—amylase; CHOL—cholesterol; ALT—alanine aminotransferase; TBIL—total bilirubin; ALP—alkaline phosphatase; CRE—creatinine; BUN/CRE—ratio between urea and creatinine; and CK—creatine kinase. *n* = 3.

**Table 7:** Hematological parameters of European catfish blood from a recirculating aquaculture system and earthen pond.

Parameter	RAS	Earthen Pond	Independent *t* test	Mann—Whitney U
WBC 10^9^/L	47.20 ± 4.91	10.83 ± 2.23	0.021	-
LYM 10^9^/L	7.76 ± 1.87	2.53 ± 0.42	0.112	-
MON 10^9^/L	2.47 ± 0.82	0.06 ± 0.01	0.099	-
NEU 10^9^/L	36.88 ± 5.98	8.03 ± 1.87	0.044	-
EOS 10^9^/L	0.09 ± 0.04	0.22 ± 0.05	0.184	-
BAS 10^9^/L	0.00 ± 0.00	0.01 ± 0.01	-	1
LYM %	17.05 ± 5.75	23.50 ± 1.00	-	1
MON %	5.10 ± 1.20	0.50 ± 0.10	0.062	-
NEU %	77.65 ± 4.65	73.75 ± 2.05	0.523	-
EOS %	0.20 ± 0.10	2.15 ± 0.95	0.178	-
BAS %	0.00 ± 0.00	0.05 ± 0.05	-	1
RBC 10^12^/L	1.01 ± 0.03	1.05 ± 0.01	-	0.33
HGB g/dL	8.40 ± 0.10	9.55 ± 0.05	0.009	-
HCT %	13.73 ± 0.50	13.70 ± 1.11	0.985	-
MCV fl	136.00 ± 1.00	131.50 ± 11.50	0.734	-
MCH pg	83.20 ± 1.50	91.50 ± 0.50	0.034	-
MCHC g/dL	61.35 ± 1.45	70.20 ± 5.80	0.277	-
RDWc %	11.55 ± 0.25	10.00 ± 0.60	0.140	-
RDWs fl	64.05 ± 1.55	56.65 ± 7.45	0.433	-
PLT 10^9^/L	24.50 ± 3.50	26.50 ± 6.50	0.812	-
MPV fl	8.20 ± 0.00	7.80 ± 0.50	-	1
PCT %	0.02 ± 0.00	0.02 ± 0.01	1.000	-
PDWc %	27.25 ± 4.15	27.85 ± 0.55	0.899	-
PDWs fl	7.60 ± 2.20	7.60 ± 0.30	1.000	-

WBC—white blood cell count; LYM—lymphocyte count; MON—monocyte count; NEU—neutrophil count; EOS—eosinophil count; BAS—basophil count; LYM—lymphocyte percentage; MON—monocyte percentage; NEU—neutrophil percentage; EOS—eosinophil percentage; BAS—basophil percentage; RBC—red blood cell count; HGB—hemoglobin concentration; HCT—hematocrit percentage; MCV—mean corpuscular volume; MCH—mean corpuscular hemoglobin; MCHC—mean corpuscular hemoglobin concentration; RDWc—red cell distribution width coefficient of variation; RDWs—red cell distribution width standard deviation; PLT—platelet count; MPV—mean platelet volume; PCT—plateletcrit percentage; PDWc—platelet distribution width coefficient of variation; and PDWs—platelet distribution width standard variation. *n* = 2.

## Data Availability

All the data that resulted from this study can be found in this paper.

## References

[1] Eurostat Database. https://ec.europa.eu/eurostat/web/fisheries/data/database.

[2] Linhart O. (2002). The culture of the European catfish, *Silurus glanis*, in the Czech Republic and in France. Aquat. Living Resour..

[3] Linhart O., Billard R., Kouřil J., Hamáčková J. (1997). Artificial insemination and gamete management in European catfish *(Silurus glanis* L.). Pol. Arch. Hydrobiol..

[4] Brzuska E., Adamek J. (1999). Artificial spawning of European catfish, *Silurus glanis* L.: Stimulation of ovulation using LHRH-a, Ovaprim and carp pituitary extract. Aquac. Res..

[5] Linhart O., Billard R. (1994). Spermiation and sperm quality of European catfish (*Silurus glanis* L.) after GnRH implantation and injection of carp pituitary extracts. J. Appl. Ichthyol..

[6] Linhart O., Billard R. (1995). Survival of ovulated oocytes and ova in the European catfish (*Silurus glanis*) after in vivo and in vitro storage or exposure to various solutions. Aquat. Living Resour..

[7] Galina I., Pronina A., Petrushin B. (2019). Techniques for in vivo extraction of gonads of male European catfish (*Silurus glanis*) for the artificial reproduction. AACL Bioflux.

[8] Bokor Z., UrbÃ¡nyi B., Horvath L., Horvath Ã. (2009). Commercial-scale cryopreservation of wels catfish (*Silurus glanis*) semen. Aquac. Res..

[9] Prokes M., Barus V., Penaz M., Hamackova J., Kouril J. (1999). Larval development and growth of the European Wels (*Silurus glanis*) under experimental conditions fed natural and pelleted diets. Czech J. Anim. Sci..

[10] Szabó T., Radics F., Borsos A., Urbányi B. (2015). Comparison of the Results from Induced Breeding of European Catfish (*Silurus glanis* L.) Broodstock Reared in an Intensive System or in Pond Conditions. Turk. J. Fish. Aquat. Sci..

[11] Bogut I., Bukvic Z., Steiner Z., Milakovic Z., Stevic I. (1998). Influence of linolenic fatty acid additive on Europan sheatfish (*Siluris glanis*) growth bred in cages. Czech J. Anim. Sci..

[12] Siwicki A.K., Pozet F., Morand M., Volatier C., Terech-Majewska E. (1999). Effects of iridovirus-like agent on the cell-mediated immunity in sheatfish (*Silurus glanis*)—An in vitro study. Virus Res..

[13] Hallier A., Serot T., Prost C. (2007). Influence of rearing conditions and feed on the biochemical composition of fillets of the European catfish (*Silurus glanis*). Food Chem..

[14] Fauconneau B., Laroche M. (1996). Characteristics of the flesh and quality of products of catfishes. Aquat. Living Resour..

[15] Geri G., Poli B.M., Gualtieri M., Lupi P., Parisi G. (1995). Body traits and chemical composition of muscle in the common carp (*Cyprinus carpio* L.) as influenced by age and rearing environment. Aquaculture.

[16] Martin J.F., Poli J.M. (1995). Etude des composantes de la qualite’ de la chair du silure glane (*Silurus glanis* L.) 2. Examen sensoriel. Piscic. Fr..

[17] Serot T., Regost C., Prost C., Robin J., Arzel J. (2001). Effect of dietary lipid sources on odour-active compounds in muscle of turbot (*Psetta maxima*). J. Sci. Food Agric..

[18] EUMOFA—European Market Observatory for Fisheries and Aquaculture Products, Recirculating Aquaculture Systems. https://www.eumofa.eu/new-eumofa-study-recirculating-aquaculture-systems.

[19] Ebeling J.M., Timmons M.B., Tidwell J. (2012). Recirculating Aquaculture Systems. Aquaculture Production Systems.

[20] Bi B., Yuan Y., Zhao Y., He M., Song H., Kong L., Gao Y. (2023). Effect of crowding stress on growth performance, the antioxidant system and humoral immunity in hybrid sturgeon. Aquac. Rep..

[21] Piazzon M.C., Naya-Català F., Perera E., Palenzuela O., Sitjà-Bobadilla A., Pérez-Sánchez J. (2020). Genetic selection for growth drives differences in intestinal microbiota composition and parasite disease resistance in gilthead sea bream. Microbiome.

[22] Burducea M., Dincheva I., Dirvariu L., Oprea E., Zheljazkov V.D., Barbacariu C.-A. (2022). Wheat and Barley Grass Juice Addition to a Plant-Based Feed Improved Growth and Flesh Quality of Common Carp (*Cyprinus carpio*). Animals.

[23] Pruszyñski T., Pistelok F. (1999). Biological and economical evaluation of african and european catfish rearing in water recirculation systems. Arch. Pol. Fish..

[24] Hamackova J., Kouril P., Kozak Z., Stupka J. (2006). Clove oil as an anaesthetic for different freshwater fish species. Bulg. J. Agric. Sci..

[25] Boiangiu R.S., Bagci E., Dumitru G., Hrițcu L., Todirașcu-Ciornea E. (2023). Promnesic, anxiolytic and antioxidant effects of *Glaucosciadium cordifolium* (Boiss.) Burtt & Davis essential oil in a zebrafish model of cognitive impairment. Plants.

[26] Bradford M.M. (1976). A rapid and sensitive for the quantitation of microgram quantities of protein utilizing the principle of protein-dye binding. Anal. Biochem..

[27] https://www.fao.org/3/v7180e/V7180E0j.htm.

[28] Lazar M., Miron L., Gostin I., Rîmbu C., Lazar R., Guguianu E. (2014). Investigations in associated protozoa-bacterial infections of cyprinids from a fish farm situated on the Jijia river in N-E of Romania. Arq. Bras. Med. Vet. Zootec..

[29] Barbacariu C.-A., Rimbu C.M., Dirvariu L., Burducea M., Boiangiu R.S., Todirascu-Ciornea E., Dumitru G. (2022). Evaluation of DDGS as a Low-Cost Feed Ingredient for Common Carp (*Cyprinus carpio* Linneus) Cultivated in a Semi-Intensive System. Life.

[30] Mongirdas V., Kusta A. (2006). Oxygen mass balance in a recirculation aquaculture system for raising European Wels (*Silurus glanis* L.). Ekologija.

[31] Zdenek A., Grecu I., Metaxa I., Sabarich L., Blancheton J.P. (2015). Processing traits of European catfish (*Silurus glanis* Linnaeus, 1758) from outdoor flow-through and indoor recycling aquaculture units. J. Appl. Ichthyol..

[32] Dokuchaeva S.I. (2006). Technology of European catfish (*Silurus glanis* L.) cultivation in pond fish farms of the Republic of Belarus. Vestsi Natsyyanal’naj akadehmii navuk Belarusi. Seryya agrarnykh navuk (Belarus). Proceedings of the National Academy of Sciences of Belarus. Agrar. Ser..

[33] Jankowska B., Zakes’ Z., Zmijewski T., Ulikowski D., Kowalska A. (2007). Slaughter value and flesh characteristics of European catfish (*Silurus glanis*) fed natural and formulated feed under different rearing conditions. Eur. Food Res. Technol..

[34] Rastiannasab A., Afsharmanesh S., Rahimi R., Sharifian I. (2016). Alternations in the liver enzymatic activity of Common carp, *Cyprinus carpio* in response to parasites, *Dactylogyrus* spp. and *Gyrodactylus* spp.. J. Parasit. Dis..

[35] Hastuti S., Subandiyono S., Windarto S. (2019). Blood performance of jaundice catfish *Clarias gariepinus*. AACL Bioflux.

[36] Rojas V., Morales-Lange B., Avendaño-Herrera R., Poblete-Morales M., Tapia-Cammas D., Guzmán F., Marshall S.H., Mercado L. (2018). Detection of muscle-specific creatine kinase expression as physiological indicator for Atlantic salmon (*Salmo salar* L.) skeletal muscle damage. Aquaculture.

[37] Docan A., Cristea V., Grecu I., Dediu L. (2010). Haematological response of the European catfish, *Silurus glanis* reared at different densities in “flow-through” production system. Arch. Zootech..

[38] Ren Q., Wang X., Li W., Wei Y., An D. (2020). Research of dissolved oxygen prediction in recirculating aquaculture systems based on deep belief network. Aquac. Eng..

[39] Khater E.S., Bahnasawy A., El-Ghobashy H., Shaban Y., Elsheikh F., El-Reheem S.A., Aboegela M. (2021). Mathematical model for predicting oxygen concentration in tilapia fish farms. Sci. Rep..

[40] Bayir A., Necdet Sirkecioglu A., Bayir M., Haliloglu H.I., Aksakal E., Günes M., Aras N.M. (2011). Influence of season on antioxidant defense systems of *Silurus glanis* Linnaeus (*Siluridae*) and *Barbus capito capito* Güldenstädt (*Cyprinidae*). Fresenius Environ. Bull..

[41] Artenie M.A., Olteanu Z., Oprică L., Bălan M. (2010). Researches on the activity of oxidoreductases from tissues sampled in different stages of development at *Silurus glanis*. Lucr. Ştiinţ. Ser. Zooteh..

[42] Obirikorang K.A., Acheampong J.N., Duodu C.P., Skov P.V. (2020). Growth, metabolism and respiration in Nile tilapia (*Oreochromis niloticus*) exposed to chronic or periodic hypoxia. Comp. Biochem. Physiol. Part A Mol. Integr. Physiol..

[43] Regoli F., Gorbi S., Frenzilli G., Nigro M., Corsi I., Focardi S., Winston G.W. (2002). Oxidative stress in ecotoxicology: From the analysis of individual antioxidants to a more integrated approach. Mar. Environ. Res..

[44] Chowdhury S., Saikia S.K. (2020). Oxidative Stress in Fish: A Review. J. Sci. Res..

[45] Fatima M., Ahmad Y., Sazeed Z., Athar M., Raisuddin S. (2000). Pollutant-induced over-activation of phagocytes is concomitantly associated with peroxidative damage in fish tissues. Aquat. Toxicol..

[46] Birnie-Gauvin K., Costantini D., Cooke S.J., Willmore W.G. (2017). A comparative and evolutionary approach to oxidative stress in fish: A review. Fish Fish..

[47] Rueda-Jasso R., Conceic L.E.C., Dias J., De Coen W., Gomes E., Rees J.F., Soares F., Dinis M.T., Sorgeloos P. (2004). Effect of dietary nonprotein energy levels on condition and oxidative status of Senegalese sole (*Solea senegalensis*) juveniles. Aquaculture.

[48] Hoseinifar S.H., Yousefi S., Van Doan H., Ashouri G., Gioacchini G., Maradonna F., Carnevali O. (2021). Oxidative Stress and Antioxidant Defense in Fish: The Implications of Probiotic, Prebiotic, and Synbiotics. Rev. Fish. Sci. Aquac..

[49] Li M., Kong Y., Wu X., Guo G., Sun L., Lai Y., Zhang J., Niu X., Wang G. (2022). Effects of dietary curcumin on growth performance, lipopolysaccharide-induced immune responses, oxidative stress and cell apoptosis in snakehead fish (*Channa argus*). Aquac. Rep..

[50] EFSA (2005). “*Clostridium* spp. in foodstuffs”—Opinion of the Scientific Panel on Biological Hazards on the request from the Commission related to *Clostridium* spp. in foodstuffs. EFSA J..

[51] Robles S., Rodrigues J., Granados I., Guerrero M.C. (2000). Sulphite-reducing clostridia in the sediment of a high mountain lake (Laguna Grands, Gredos, Spain) as indicators of fecal pollution. Internat. Microbiol..

[52] (1998). Council Directive 98/83/EC of 3 November 1998 on the Quality of Water Intended for Human Consumption.

[53] (2013). Water Quality—Enumeration of *Clostridium perfringens*—Method Using Membrane Filtration (ISO 14189:2013).

[54] (2014). Water Quality—Enumeration of *Escherichia coli* and Coliform Bacteria—Part 1: Membrane filtration Method for Waters with Low Bacterial Background Flora (ISO 9308-1:2014).

[55] Frick C., Vierheilig J., Linke R., Savio D., Zornig H., Antensteiner R., Baumgartner C., Bucher C., Blaschke A.P., Derx J. (2018). Poikilothermic animals as a previouslyunrecognized source of fecal indicator bacteriain a backwater ecosystem of a large river. Appl. Environ. Microbiol..

[56] Zhang P. (2002). Influence of Foods and Nutrition on the Gut Microbiome and Implications for Intestinal Health. Int. J. Mol. Sci..

[57] Abdul Razak S., Bauman J.M., Marsh T.L., Scribner K.T. (2022). Changes in Lake Sturgeon Gut Microbiomes Relative to Founding Origin and in Response to Chemotherapeutant Treatments. Microorganisms.

[58] Acosta M., Quiroz E., Tovar-Ramírez D., Roberto V.P., Dias J., Gavaia P.J., Fernández I. (2022). Fish Microbiome Modulation and Convenient Storage of Aquafeeds when Supplemented with Vitamin K1. Animals.

[59] Simeanu C., Măgdici E., Păsărin B., Avarvarei B.-V., Simeanu D. (2022). Quantitative and Qualitative Assessment of European Catfish (*Silurus glanis*) Flesh. Agriculture.

